# A Pharmacovigilance Study in Medicine Department of Tertiary Care Hospital in Chhattisgarh (Jagdalpur), India

**DOI:** 10.4103/0975-1483.62222

**Published:** 2010

**Authors:** H Singh, N Dulhani, BN Kumar, P Singh, P Tewari, K Nayak

**Affiliations:** *Department of Pharmacology, Government Medical College, Jagdalpur, Chhattisgarh, India*; 1 *Department of Medicine, Government Medical College, Jagdalpur, Chhattisgarh, India*; 2 *Department of Anatomy, Government Medical College, Jagdalpur, Chhattisgarh, India*; 3 *Department of Pharmacology, Government Medical College, Rewa, MP, India*

**Keywords:** Pharmacovigilance, adverse drug reaction, polypharmacy

## Abstract

The aim of the present study was to observe adverse drug reactions (ADRs) with respect to polypharmacy at tertiary care centre at Bastar, Jagdalpur (Government Medical College, Jagdalpur). A prospective, observational evaluation of the ADRs conducted over a period of 6 months in Department of Medicine in Government Medical College, Jagdalpur. During the study period, a total of about 4850 patients visited the OPD and inpatient ward of medicine department, and 154 ADRs events were reported. Out of 154 reports that were identified, a higher percentage of ADRs in females (51.29%) was observed as compared to males (48.7%). Of the 154 ADRs, 76 (49.35%) were found to be mild, 55 moderate (35.71%), and 23 severe (14.93%). A total of 99 (64.28%) ADRs were observed in patients receiving four or more medications concurrently. Conversely 55 (35.71%) ADRs were detected in patients using three or less medicines. The largest number of reports was associated with antimicrobial therapy (28.57%), followed by antihypertensive (24.02%) and antidiabetics (14.28%). Among the affected organ systems, gastrointestinal ADRs constituted a major component (39.61%) followed by skin reactions (28.57%). On causality assessment, nearly 36.36% ADRs were considered as probable, 31.16% possible, and 9.74% could not be categorized and were placed under unassessable. Expected, limited ADR are permissible in normal clinical setting, but the present study focuses on the result showing increased and amplified ADR associated with the polypharmacy practices, which may be curtailed with rational drug prescribing habit.

## INTRODUCTION

ADR monitoring and reporting helps in detection and prevention of reoccurrence of ADRs. The objective of this study was to conduct ADR reporting in department of General Medicine, Government Medical College, Jagdalpur, Chhattisgarh, India.

According to WHO’S definition an Adverse Drug Reaction (ADR) is a response to a drug that is noxious and unintended and occurs at doses normally used in human for the prophylaxis, diagnosis, and treatment of disease, or for modification of physiological function.[1,2]

The factors such as polypharmacy, age, gender, race, genetics, multiple, and inter-current diseases can cause morbidity and mortality. Lazarous *et al*. estimated that ADRs were the fourth to sixth largest cause of death in the United States.[3] In United Kingdom most of the studies were performed in the previous two decades and were restricted to specific areas such as monitoring of ADRs in geriatric patients.[4–11] The largest UK study was based on retrospective review of case reports and gave poor documentation.[12]

The detection of adverse drug reactions (ADRs) has become increasingly significant because of introduction of a large number of potent toxic chemicals as drugs in the last two or three decades. WHO has intervened seriously in this matter and established an international adverse drug reactions monitoring centre at Uppsala, Sweden, which is collaborating with national monitoring centers in around 70 countries.[1]

In many countries, drug utilization studies have been performed by means of prescription databases, such as the Tayside database in Scotland,[13] the VAMP database in England,[14] the Saskatchewan database in Canada,[15] the Compass and the Kaiser Permanente databases in USA,[16] and the Pharma database in the Netherlands.[17] Generally, prescription databases have proved their value as powerful research tools for a multitude of pharmacoepidemiological studies, and studies comparing drug exposure according to different sources have demonstrated registered data as one of the most accurate sources of data.

As per the ADR monitoring in our country, very little attention has been given so far and very few original studies have been done in this regard. We have very few ADR monitoring centers right now and lot of efforts is required in order to collect ADR data which may generate safety surveillance of billions of therapeutically active substances either alone or in combinations.

Avoidable adverse effects will be reduced by more skilful prescribing and this means that doctors, among all the other claims on their time, must find time to understand drugs better, as well as to understand their patients and their diseases.[18]

Taking this view in mind we would like to strengthen the ADR monitoring and spreading awareness among our clinicians, we performed a prospective, observational analysis of ADRs caused by medicines prescribed in the department of Medicine with the help of department of Pharmacology in Government Medical College, Jagdalpur to identify problem prevalence and to assess causality of these reactions.

## MATERIALS AND METHODS

Study of ADR monitoring was prospective, observation study done from July 2008 to December 2008 over six month’s duration in Medicine department with help of department of Pharmacology. An informed consent was taken from the patients who experienced ADRs for participating in the study. The study was initiated after the approval of the study protocol by Institutional Review Board.

### Inclusion criteria

The inclusion criteria of this study were all the suspected ADRs that may be due to the medications, both prescribed and over the counter, taken by patients either as inpatients or outpatients, that were ultimately noted and reported.

### Exclusion criteria

- The use of alternative system of medicines like Ayurveda, Homeopathy, Unani, etc.
- Over prescribing, over dosage, and excess consumption
- Patients taking more than ten prescription drugs
- All mentally retarded
- Drug addicts
- Unconscious and patients unable to respond to verbal questions were also excluded from the study.

#### Preparation of adverse drug reaction reporting forms (yellow cards)

Yellow cards were prepared which included all the relevant data such as name of the patient, age, sex, height, weight, date of occurrence of events, brief description of the reaction, name of the suspected drug causing the reaction, duration of reaction, and name of the clinician reporting the reaction on the basis of WHO guidelines.

### Collection of data

Upon receiving the report, investigator visited the respective ward or the department and collected the necessary details. When an ADR was suspected, the data from the patient profile form such as patient details, patient medication details including non-prescription drugs, alternative treatments and recently ceased medications, comprehensive adverse reaction details including description of the reaction, time of onset and duration of the reaction and treatment given with relevant investigation reports were collected.

Causality and severity assessment: The causality was assessed by using Naranjo causality assessment scale and the severity was assessed by using the Hartwig severity assessment scale according to the recommendation by the WHO Uppsala Monitoring Center.[12] Data extracted independently by two investigators were analyzed by a random-effects model. To obtain the overall incidence of ADRs in hospitalized patients, we combined the incidence of ADRs occurring while in the hospital plus the incidence of ADRs causing admission to hospital. We excluded errors in drug administration, noncompliance, overdose, drug abuse, therapeutic failures, and possible ADRs. Serious ADRs were defined as those that required hospitalization, were permanently disabling, or resulted in death. To confirm adverse drug reactions, if some investigations were required, they were carried out with the consent of the concerned physician.

### Statistical method

From pooled data we calculated mean, standard deviation, percentage, and as required the Chi-squared (**χ**^2^) test were applied to find the association between outcome and parameters and *P*-values less than 0.05 were considered as significant.

## RESULTS

During the 6 months study period, a total 4850 patients visited the medicine OPD and inpatients. The demographic data is as follows; among 4850 patients 3150 were in age group of less than 35 years and 1700 in age group of more than 35 years [Table 1]. Total male subjects were 2986 and females were 1864. A total of 154 ADRs (3.17%) were reported in 4850 patients.

**Table 1:** Demographic data of patients who had adverse drug reactions

Demographic parameter	No. of ADRs (%)
Age wise	
<35 years	84 (54.5)
>35 years	70 (45.45)
Sex wise	
Male	75 (48.71)
Female	79 (51.29)

The gender distribution among the patients, who experienced ADRs were 75 (48.71%) males and 79 (51.29%) females. Taking the whole study population (4850) females (1864) have experienced more number of ADRs as compared to the male (2986) population. Similarly, among 4850 study population, 3150 were less than 35 years and 1700 patients were more than 35 years age group, and they had 84 patients (54.55%) and 70 patients (45.5%) with ADRs, respectively. Their statistical significance is shown in Table 2.

**Table 2:** Statistically significant correlation of adverse drug reactions with age and sex of patients

Demographic data	Total no. of patient’s in study	No. of patients with ADRs (%)	Chi-square test result
Age			
<35years	3150	84 (54.55)	*P* = 0.006 < 0.05 significant (with df = 1, and confidence level = 95%)
>35 years	1700	70 (45.5)	
Total	4850	154	
Sex			
Male	2986	75 (48.71)	*P* = 0.001 < 0.05 significant (with df = 1, and confidence level = 95%)
Female	1864	79 (51.29)	
Total	4850	154	

As expected, Polypharmacy had a major influence on the occurrence of ADRs with a total of 99 (64.28%) ADRs observed in patients receiving four or more medications concurrently [Table 3]. Conversely, 55 (35.71%) ADRs were detected in patients on three or less medicines. The frequency of ADRs associated with different routes of administration was as follows: Oral (n = 135), parentral (n = 16), and topical (n = 3) [Table 4].

**Table 3:** Number of drugs and adverse drug reactions (%)

No. of drugs	No. of ADRs (%)	(%)
1	14 (9.09)	55 (35.71)
2	14 (9.09)	
3	27 (17.53)	

4	22 (14.28)	99 (64.28)
5	32 (20.78)	
6	20 (12.99)	
7	25 (16.23)	
Total	154	

**Table 4:** Adverse drug reaction related to route of drug administration

Route	ADRs (%)
Oral	135 (87.66)
Parentral	16 (10.39)
Topical (local)	3 (1.95)
Total	154

The gastrointestinal side effects (e.g. gastritis, dysphasia, etc.) were at the top with 39.61% followed by skin, and subcutaneous disorders (28.57%). Other main groups were respiratory 17 (11.03%), CNS and neurological disorders (8.44%). The detailed description of organ systems affected by ADRs is shown in Table 5.

**Table 5:** Adverse drug reaction and organ system involved

Organ system involved	No. of ADRs (%)
Gastrointestinal disorders	61 (39.61)
Skin, mucous membrane	44 (28.57)
Respiratory disorders	17 (11.03)
CNS and neurological disorders	13 (8.44)
Blood and lymphatic system	5 (3.25)
Hepato-billiary disorders	2 (1.29)
Others	12 (7.79)
Totals	154

Out of a total number of 154 ADRs, 15 (9.74%) were classified as certain, e.g. hypersensitivity reaction with intravenous contrast medium, skin reaction with cefotaxime injection, itching and dermatitis with etophylline tablets, and hypoglycemia with glibenclamide tablets. 56 ADRs (36.36%) were considered probable e.g. dry cough with enalapril and dysphasia with furosemide tablets. 48 (31.16%) were classified as possible e.g. loss of appetite and pain in abdomen with antitubercular medicines and breathlessness with nimesulide and metoprolol. 15 ADRs (9.74%) could not be categorized and were placed under unassessable category e.g. itching with antitubercular drugs and mental depression with metoprolol [Table 6].

**Table 6:** Causality assessment of adverse drug reactions

Probability scale	No. of ADRs (%)
Certain	15 (9.74)
Probable	56 (36.36)
Possible	48 (31.16)
Unlikely	08 (5.19)
Conditional	12 (7.79)
Un-assessable	15 (9.74)
Total	154

Out of the 154 ADRs, 76 (49.35%) were found to be mild e.g. cold extremities with atenolol, 55 (35.71%) moderate e.g. dry cough with ramipril, and 23 (14.93%) severe e.g. subcutaneous bleeding with carbimazole. Most of the severe ADRs were associated with antitubercular, oral hypoglycemic drugs, insulin, and heparin. These were reported more commonly with injectables as compared to oral medications. Ten serious and life-threatening ADRs were also reported e.g. hepatitis with antitubercular medicines and anaphylactic reactions with iopromide (i.v. contrast medium). Drug-induced morbidity is an important cause of hospitalization and is associated with significant mortality. Most of the ADRs observed in our study were either mild or moderate [Table 7].

**Table 7:** Classification of adverse drug reactions on the basis of severity

Severity	No. of ADRs (%)
Mild	76 (49.35)
Moderate	55 (35.71)
Severs	23 (14.93)
Total	154

Distribution of ADRs across therapeutic classes was as follows: Antimicrobials (28.57%), antihypertensives (24.02%), antidiabetics (14.28%), and NSAIDs (9.74%) [Table 8]. Among the individual drugs, ramipril was associated with maximum cases of ADRs (6.6%) followed by amlodipine (5.7%) and atenolol (4.1%). In case of fixed dose drug combinations, isoniazid + rifampicin + ethambutol + pyrazinamide combination was responsible for 13.1% ADRs.

**Table 8:** Pharmacological classes of drugs implicated to cause adverse drug reactions

Drug classes	No. of ADRs (%)
Antimicrobials	44 (28.57)
Antihypertensive	34 (24.02)
Anti-diabetics	22 (14.28)
NSAID	15 (9.74)
Blood and blood products	14 (9.09)
CNS drugs	11 (7.14)
Anticoagulants	6 (3.89)
Others	8 (5.19)
Total	154

The statistical significant difference was observed as more ADR found in age group of more than 35 years and more ADR in female subjects as compared to age group less than 35 years and male subjects, respectively [Table 2].

## DISCUSSION

World Health Organization (WHO) under the Pharma covigilance program recruited about 78 countries as its members.[19,20] Most of these member countries have a well-established ADR reporting system and primarily doctors are given responsibility to report ADRs. In India, the National Pharmacovigilance Program (NPP) encourages the doctors and hospital pharmacists to report ADRs.

The primary objective was to estimate the prevalence of the ADR; in secondary objective we examined the different factors that influence the outcome such as age, sex, the effect of Polypharmacy, and effects of route of administration of drugs.

Although female population had more prevalence of ADRs, the difference was significant with respect to the male population. Previous studies also reported that the occurrence of ADRs is more common in women.[21] this finding may be because of the differences in weight and body mass index, hormonal changes unique to females (during puberty, menstrual cycles, menopause), and the effect of these changes on drug metabolism. Other possible factors include differences in fat composition (with respect to impact on drug distribution) and genomic constitutional differences influencing the levels of various enzymes involved in drug metabolism.[22,23]

In another study, Gor *et al*. showed overall 3% ADR, and similar to our study majority of ADRs were due to chemotherapeutic agents but in their study on sex of the patients did not influence the incidence rate of ADR, as in our study females were more prone to ADR.[24] In other study by Rajan *et al*., 0.22% ADR were reported which is very low as compared to our (3.17%) while rest of their finding are similar to our study.[25]

In our study, the majority of ADRs were in >35 years age group. The reasons that could be attributed are that the patients of this age group suffered from hypertension and diabetes. So this age group used more number of medicines and frequently visited the medicine OPD for their regular check-up and complained about drug-related adverse events, though most of these adverse events were mild and easily tolerated.

A majority of the ADRs were associated with oral administration of medicines followed by parenteral route. Most of the ADRs with injectable medications were severe. The three topical reactions observed was erythema (localized skin redness) on application of heparin sodium cream. Gastrointestinal ADRs were most commonly observed with oral medications.

The incidence of adverse drug events is directly proportional to the number of drugs being taken and increases remarkably as number of drugs rises. Many epidemiological studies of risk factors for adverse drug reactions have shown that the number of concurrently used drugs is the most important predictor of these complications.[26] Polypharmacy needs to be discouraged as a good number of ADRs results from drug-drug interactions.

Both hypertensive and diabetic patients are predisposed to ADRs as in our study and they are at inevitable risk of bad effects of drugs due to sub-optimal functionality of their organ systems. This necessitates careful organ function analysis prior to prescription writing for any medication. One of the essential reasons of wide prevalence of ADRs in hypertensive and diabetic patients is that they are elderly and are often on multiple-drug therapy.

In our study, we found gastrointestinal side effects (e.g. gastritis, dysphagia, etc.) at the top of the list of ADRs followed by skin and subcutaneous disorders. Next main groups are metabolic, nutritional, CNS, and neurological disorders. Neurological ADRs were at the top of the list of ADRs in previous studies, and gastrointestinal ADRs were reported among the top three groups of ADRs.[27,28]

The present study is done on quite a large number of subjects over a prolong period but the limitations were the loss of subjects during follow up and the low awareness of subjects and unintentional ignorance of adverse effects by the treating physician which may be the reason for the low ADR prevalence (3.17%).

The major controversy arising from this study is pointing toward the part of both parties: Patient and physician, the patients being unaware of the ADRs. In our hospital and other health care facilities, the documentation of ADRs is getting unintentionally missed which could be because of technical or shortage of staff or lack of proper sensitization, and many a times the mortality and morbidity associated with ADR are taken as outcome of disease processes itself.

## CONCLUSION

The present work is the maiden pharmacovigilance study conducted at our teaching hospital. It has provided baseline information about the prevalence of ADRs and their distribution among different age groups, genders, organ systems affected, and therapeutic classes of medicines. The data presented here will be useful in future, long term and more extensive ADR monitoring in the hospital and will be useful in framing policies towards rational use of drugs.

There is a need to inform the treating doctors about the importance of observing for ADR following pharma cotherapy, recording them meticulously, and reporting them to the concerned authority. This practice will prove to be very valuable in making the drug therapy safer and rational.

So in future a comprehensive sensitization Programme is required in each step of health care system right from treating doctors, nurses, paramedics, and drug dispensing pharmacist to ensure better and safe pharmacotherapy and improve compliance of patients.
